# Medical Certificates Again

**Published:** 1898-12-31

**Authors:** 


### MEDICAL CERTIFICATES AGAIN.

We have again and again alluded to the laxity with which medical certificates are often given, and we should almost apologise for drawing attention to the matter afresh were it not that medical men, sometimes no doubt while endeavouring to "act for the best," are so frequently led to exceed their duty, and to do what cannot be justified, in certifying to facts of which they are not themselves personally cognisant.

The following is published in *The Leader*, December 23rd, but we leave out the name of the medical man in question, as we see no object in pointing at an individual. It is the practice against which we protest:—

"Dr. Millson has reported to the Newington Vestry that two children had been certified by Dr. —— to be suffering from scarlet fever, and had been removed to Park Hospital. The children turned out to be quite healthy, and it has also been stated that the doctor gave the certificate without having seen the children. The mother has been fined for sending one of them to school. The vestry declined to pay Dr. —— the notification fee, and the question of the mother obtaining compensation was referred to one of the vestry committees. A reporter of the *Morning Leader* saw Dr. —— at his surgery last night. 'I contend that I acted quite rightly,' said Dr. ——. 'I was called to see a child, and found it suffering from the febrile stage of scarlatina. I was informed that there were two other children in the house who had shown practically the same symptoms, though in a milder form, some ten days or so before. It is quite true that I did not see those two children, for the mother considered there was no need for medical aid. But from what I was told I was strongly of opinion that they had had scarlet fever, and were at that moment in the convalescent stage, when the peeling takes place, and the danger of infection is greatest. If it were allowed to us, I should have certified as to the one, and added a note to the certificate mentioning my suspicion as to the others. But the vestry do not allow that. In my view the safest thing therefore was to notify all three in the usual way, and leave the vestry to take the steps it thought proper.'"

